# Persistent Hypoglossal Artery as a Rare Anatomical Variation Mimicking Carotid Artery Aneurysm: A Case Report

**DOI:** 10.7759/cureus.69206

**Published:** 2024-09-11

**Authors:** Nikola Cimbaljević, Slobodan Tanasković, Vladimir Mihajlović, Želimir Antonić, Nenad Ilijevski

**Affiliations:** 1 Vascular and Endovascular Surgery, Vascular Surgery Clinic, Institute for Cardiovascular Diseases “Dedinje”, Belgrade, SRB; 2 Faculty of Medicine, University of Belgrade, Belgrade, SRB; 3 Radiology, Clinic for Radiology, Institute for Cardiovascular Diseases “Dedinje”, Belgrade, SRB

**Keywords:** anatomic variant, carotid-vertebrobasilar anastomosis, msct angiography, persistent hypoglossal artery, vertebrobasilar insufficiency

## Abstract

We present a case of a rare vascular variation of the persistent hypoglossal artery (PHA) in a 57-year-old Caucasian female patient with a medical history of poorly controlled hypertension, headaches, diabetes mellitus, and depression. This anatomical variation was initially misdiagnosed as an internal carotid artery (ICA) aneurysm during the extracranial carotid Doppler imaging conducted due to nonspecific symptoms of cerebrovascular insufficiency, manifesting as coordination disturbances. PHA is one of the four vertebrobasilar anastomoses, originating from the cervical segment of the ICA. Together with the hypoglossal nerve, the meningeal branch of the ascending pharyngeal artery, and an emissary vein from the basilar plexus, it traverses the hypoglossal canal and enters the posterior cranial fossa, where it joins the basilar artery. This artery typically involutes during embryonic development but can persist into adulthood. If present, PHA is most often asymptomatic; however, it can be associated with pathological conditions such as atherosclerosis, cerebral ischemia, aneurysms, and arteriovenous malformations. This case highlights the diagnostic challenges posed by this anatomical variation and underscores the importance of further investigations in confirming accurate vascular anatomy. Here, we discuss the diagnostic criteria for PHA, its clinical significance, and therapeutic modalities.

## Introduction

Carotid-vertebral anastomoses are arteries that originate from the internal carotid artery (ICA) and the dorsal aorta, supplying blood to the future basilar artery and posterior circulation in early embryonic development, as first described by Padget [1]. These anastomoses encompass four arteries named after surrounding structures: trigeminal, otic, hypoglossal, and proatlantal intersegmental arteries [1]. These arteries involute and form the vertebral and posterior communicating arteries during embryonic development [2]. The persistent hypoglossal artery (PHA) is the second most common carotid-vertebral anastomosis, following the trigeminal artery [3]. PHA is most commonly asymptomatic and is discovered incidentally during radiological imaging for other conditions [4]. This anatomical variation can be involved in pathological processes such as atherosclerosis, cerebral ischemia, aneurysms, and arteriovenous malformations [5-7]. Due to potential pathological processes and complications, PHA represents not only a physiological phenomenon but also a significant medical entity that, due to its rarity, poses a challenge for diagnosis and therapy in everyday practice [4]. Treatment options for PHA include both open surgical and endovascular approaches. This case report describes a PHA that was initially misdiagnosed as an ICA aneurysm. The report will specifically address diagnostic criteria, clinical significance, and therapeutic modalities.

## Case presentation

**Figure 1:**
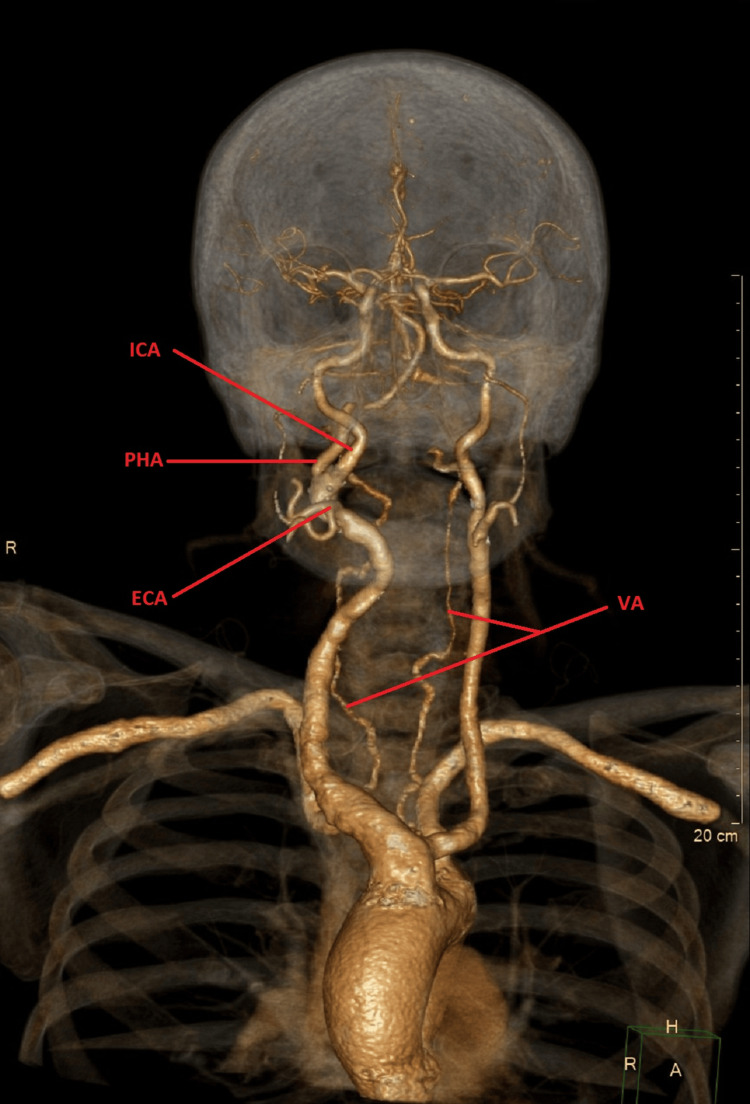
The origin of the PHA from the cervical segment of the internal carotid artery is associated with hypoplastic vertebral arteries in the cervical segment. MSCT angiography of supra-aortic branches in a 57-year-old female patient reveals the origin of persistent hypoglossal artery from the cervical segment of the internal carotid artery. The image shows hypoplasia of both vertebral arteries. MSCT: Multi-slice computed tomography; ICA: Internal carotid artery; ECA: External carotid artery; PHA: Persistent hypoglossal artery; VAs: Vertebral arteries

A 57-year-old female patient presented to the Emergency Department on two occasions due to severe headaches, high blood pressure, epistaxis, and dizziness. The patient has a history of hypertension, diabetes mellitus, and depression. She underwent a carotid Doppler ultrasound due to complaints of occasional coordination disturbances. The examination revealed a suspected aneurysm of the right ICA with a diameter of approximately 12 mm. Due to the suspicion of an ICA aneurysm, the patient was examined by a vascular surgeon and subsequently referred for multi-slice computed tomography (MSCT) angiography of the supra-aortic branches. This imaging revealed that it was not an ICA aneurysm, but rather an anomalous bifurcation and a vessel originating from the right ICA (Figure 1).

**Figure 2:**
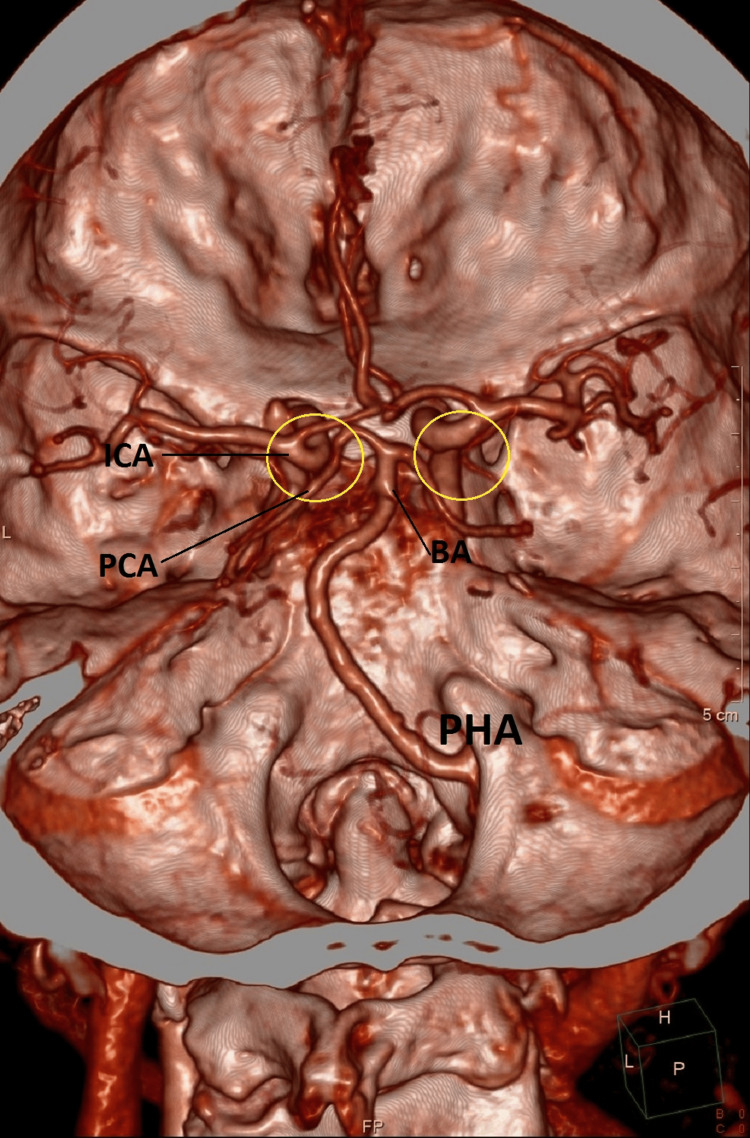
The basilar artery originates from the PHA, with aplasia of the intracranial segment of the vertebral arteries and the posterior communicating arteries. The image shows a persistent hypoglossal artery entering the posterior cranial fossa, subsequently forming the basilar artery in its onward course. There is also bilateral aplasia of the intracranial segment of the vertebral arteries and posterior communicating arteries. The posterior communicating arteries typically connect the internal carotid artery and the posterior cerebral artery, bridging the anterior and posterior cerebral circulation. When these communicating arteries are present, they are located in the region marked by the yellow circle. PHA: Persistent hypoglossal artery; BA: Basilar artery; ICA: Internal carotid artery; PCA: Posterior cerebral artery

Hypoplasia of both vertebral arteries was also observed. MSCT angiography of the intracranial segment detected that this anomalous vessel forms the basilar artery (Figure 2).

**Figure 3:**
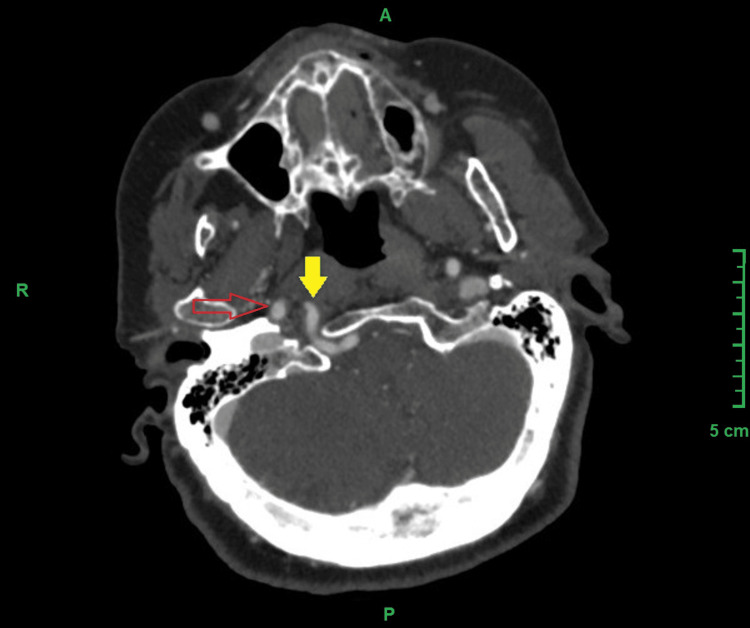
MSCT angiography, axial view, showing the persistent hypoglossal artery passing through the hypoglossal canal. Persistent hypoglossal artery, marked with a yellow arrow, and right internal carotid artery, marked with a red arrow. MSCT: Multi-slice computed tomography

Axial sections revealed that this artery enters the cranium through the hypoglossal canal (Figure 3).

Based on all these criteria, it was concluded that this is a PHA. Significant atherosclerotic changes were not observed on the angiography of the ICA and PHA. Magnetic resonance imaging (MRI) of the brain was performed, revealing only minor lacunar lesions with no evidence of larger ischemic lesions. The patient was examined by a neurologist, who assessed that the lacunar ischemic lesions were a result of small vessel disease in the brain and poorly controlled hypertension. Following the diagnostic procedures and in consultation with a vascular surgeon, neurologist, and interventional radiologist, it was deemed appropriate to continue with medical therapy due to associated comorbidities and the high branching of the PHA. At the six-month follow-up, the patient reports feeling subjectively well and notes that her symptoms are less pronounced following the initiation of neurological therapy and successful regulation of high blood pressure.

## Discussion

In early embryonic development, the hindbrain is vascularized by a pair of longitudinal neural arteries that receive blood from the primitive ICA through vertebrobasilar anastomoses [8]. There are four vertebrobasilar anastomoses, including the primitive trigeminal artery (PTA), primitive optic artery (POA), PHA, and primitive proatlantal artery (ProA) [9]. The most common is PTA, with a frequency of 0.5%. The PHA is the second most frequent carotid-vertebral anastomosis, with a frequency ranging from 0.03% to 0.09%, while POA and ProA represent extremely rare vascular variations [3]. In further embryonic development, with the formation of posterior communicating arteries, these anastomoses regress. However, if there is aberrant development of the posterior circulation, cerebrovascular anastomoses can persist into adulthood. PHA typically originates from the posterior wall of the ICA in the cervical segment between C1 and C3, although, rarely, it can also arise from the external carotid artery [4]. PHA then passes through the hypoglossal canal en route to the posterior cranial fossa, where it forms the basilar artery. The hypoglossal canal is a bilateral bony canal at the base of the skull, positioned near the occipital condyles and jugular foramen. PHA passes through this canal along with other common elements, such as the hypoglossal nerve, the meningeal branch of the ascending pharyngeal artery, and an emissary vein from the basilar plexus [10].

PHA is most commonly asymptomatic in nature and is discovered as an incidental finding in imaging studies [4]. PHA can pose challenges for Doppler sonographic examination due to its high branching, as in our case, where it was mistakenly interpreted as an aneurysm of the ICA. The misdiagnosis is influenced by the fact that it involves a rare variation and that physicians performing Doppler ultrasonography do not typically anticipate encountering this variation in their everyday practice. The definitive diagnosis is typically established using MSCT angiography, magnetic resonance angiography, or digital subtraction angiography (DSA).

Brismar has established criteria for the diagnostic identification of PHA: (I) the hypoglossal artery originates from the ICA as a robust branch at the C1-C3 levels; (II) it enters the posterior cranial fossa through the hypoglossal canal; (III) the basilar artery is filled only beyond the point of junction with the anastomosis; and (IV) the posterior communicating artery is either absent or not visible on angiography [11]. The most crucial of these criteria is the passage of the artery through the hypoglossal canal. Additionally, the vertebral arteries are usually hypoplastic bilaterally, or there is aplasia of the artery on the ipsilateral side and hypoplasia on the contralateral side [12].

Due to the aplasia of the posterior communicating artery and vertebral arteries - a common occurrence in PHA - the vascularization of the brain stem, cerebellum, and ipsilateral hemisphere entirely relies on the ICA [13]. The carotid bifurcation and the initial segment of the ICA are often sites of atherosclerotic disease. Significant stenoses in the ipsilateral ICA compromise the flow in both the anterior and posterior circulations. Therefore, it is crucial to recognize this variation before planning surgical or endovascular treatment. In our case, there was no significant stenosis of the ICA or PHA.

The PHA itself can be a site prone to significant atherosclerotic disease. The branching of the PHA has a similar morphology and hemodynamics to the carotid bulb, making the PHA itself a suitable site for significant atherosclerotic disease development [5].

Given the rare occurrence of PHA, experiences in treating atherosclerotic stenoses associated with this anatomical variation are mainly presented in the literature in the form of case reports [13,14-16]. Carotid endarterectomy is successfully applied in the prevention of ischemic lesions, while, in recent times, there is an increasing use of ACI and PHA stenting. In the literature, carotid endarterectomies with [13] and without the use of a shunt [15] have been described. The decision regarding shunt usage was made intraoperatively based on stump pressure values. If the stump pressure is low (less than 40 mmHg), a shunt can be placed in the ICA and/or PHA [13]. Carotid endarterectomy can be technically challenging due to the high origin of the PHA. In such cases, carotid artery stenting (CAS) can be the method of choice in treatment. In the context of endovascular treatment, it is crucial to implement adequate prevention of distal embolization for both the anterior and posterior circulations [16].

Additionally, patients with this anatomical variation often have associated cerebral aneurysms and arteriovenous malformations [6,7].

## Conclusions

In summary, PHA represents intriguing vascular variations with potential clinical implications. In the context of atherosclerotic disease, PHA may be prone to significant pathology, mirroring the carotid bulb's characteristics. In the absence of posterior communicating and vertebral arteries, recognition of this variation becomes paramount before planning surgical or endovascular interventions, considering potential implications on both anterior and posterior cerebral circulation. Diagnostic criteria, such as its passage through the hypoglossal canal, aid in accurate identification using imaging modalities like MSCT or MR angiography.

On the other hand, ultrasonic recognition of the PHA poses a challenge due to its high separation from the ICA. Additionally, the rarity of this variation in everyday practice, coupled with the mentioned difficulty in visualization, can lead to the misidentification of the PHA as more common pathological changes, such as an aneurysm of the ICA, as was the case in our situation. Physicians performing ultrasound examinations of the neck arteries should be aware of this variation, and a definitive diagnosis should be made after MSCT or MR angiography through well-defined criteria for PHA.
